# Editorial: Applied computational social sciences

**DOI:** 10.3389/fdata.2025.1605788

**Published:** 2025-05-22

**Authors:** Paolo Parigi, Kinga Makovi

**Affiliations:** ^1^Facebook, Menlo Park, CA, United States; ^2^Social Science Division, New York University Abu Dhabi, Abu Dhabi, United Arab Emirates

**Keywords:** computational social science (CSS), tech companies, cultural construction, ethical questions, equity, social media platform, political process

This Research Topic examines the social transformations brought about by novel tools of social interactions. Man builds tools to change the world and in turn, the tools change the man that built them. It is this dynamic process that each of the articles presented here explores.

Some common themes emerge across all the contributions:

- Emphasis on the social and cultural construction of data-driven domains: Brandt examines the cultural construction of data science through the lens of motives, meaning-making, and disputes on social media. Kaul and Mukherjee focus on the “Equitable Differential Privacy” framework, arguing that ensuring equitable outcomes requires not just algorithmic design but also inclusive communication that considers diverse social groups and power dynamics. Sakamoto et al. analyze how the established practice of offline diplomacy is being augmented and selectively adopted in the online sphere, highlighting strategic and presentational considerations that reflect social norms and diplomatic goals.
- Leveraging computational methods for social inquiry: each article employs computational social science methodologies to analyze large datasets. Brandt utilizes network analysis and topic modeling on a large corpus of tweets. Kaul and Mukherjee conduct a descriptive case study, drawing on secondary sources related to the U.S. Census Bureau's communication strategies, which implicitly involves analyzing textual data and communication patterns. Sakamoto et al. explicitly use quantitative text analysis tools like word embeddings, topic modeling (LDA), and sentiment analysis on corpora of UN speeches and X posts.
- Ethical and equity concerns: two of the articles touch upon ethical and equity considerations within their respective domains. Brandt notes the emergence of concerns with “new practical and ethical standards” in the construction of data science. Kaul and Mukherjee make “equity” the central focus of their work on differential privacy, arguing for a framework that addresses the needs of all social groups, particularly marginalized ones, through both algorithmic design and inclusive communication.
- Examining the role of online platforms in professional and political practices: all three articles consider the significance of online platforms in transforming professional and political practices. Brandt uses Twitter to study the cultural construction of data science. Kaul and Mukherjee analyze communication related to the Census Bureau's DP implementation, which includes online dissemination of information. Sakamoto et al. directly compare offline diplomatic speeches with online posts on X, highlighting how diplomats leverage these platforms to complement their traditional roles.

A goal of this Research Topic is to promote the circulation of ideas between academia and tech companies by encouraging research on topics that are relevant for both communities. We think that the topics covered in this Research Topic offer a starting point for starting a dialogue between the two communities of researchers. As such, we see this as a beginning, a first step that recognizes the existence of a new domain of knowledge that uses social science concepts and ideas for building tools that shape interactions.

